# Concurrent Pancreatic and Sartorius Muscle Lipomas: A Case Report

**DOI:** 10.1002/ccr3.70350

**Published:** 2025-04-25

**Authors:** Om Prakash Bhattaa, Shritik Devkotab, Arun Kalikotec, Prakash Gyawalid, Sachin Awasthie

**Affiliations:** ^1^ Department of Internal Medicine Nova Hospital Dhangadhi Nepal; ^2^ Department of Radiodiagnosis and Imaging Postgraduate Institute of Medical Education and Research Chandigarh India; ^3^ Rani Primary Health Centre Biratnagar Nepal; ^4^ Sukraraj Tropical Infectious Hospital Kathmandu Nepal; ^5^ Department of Emergency Medicine Yashoda Hospital Banke Nepal

**Keywords:** intramuscular, lipoma, pancreas, sartorius

## Abstract

We present an incidentally diagnosed case of concurrent pancreatic and Sartorius muscle lipoma in a 65‐year‐old female with cholelithiasis. Such dual presentation has seldom been mentioned in the literature and requires thorough evaluation to differentiate it from other pathologies.

## 1 Introduction

Lipomas are common benign tumors of adipose tissue that typically occur in subcutaneous regions. Epithelial tumors make up most of the pancreatic tumors, with mesenchymal tumors accounting for 1%–2% of pancreatic tumors [1]. Pancreatic lipomas are even rarer and are diagnosed incidentally, as they are usually asymptomatic. Intramuscular lipomas are equally rare, accounting for less than 1% of all lipomas. The concurrent existence of pancreatic and sartorius muscle lipomas is rare and has not been reported in the literature.

Routine use of imaging and the familiarity of radiologists with this condition can lead to an increased rate of diagnosis. The concurrent occurrence of pancreatic and sartorius muscle lipomas in a single patient is unusual.

This case report highlights the importance of comprehensive evaluation and differentiation from other pathologies, contributing to the limited literature on these rare occurrences, and presents the case of a 65‐year‐old female who was investigated for abdominal pain following blunt abdominal trauma and was found to have an incidental pancreatic lipoma on ultrasonography(USG). Further, Computed Tomography (CT) imaging revealed a concurrent sartorius muscle lipoma.

## 2 Case Presentation

The patient was a 65‐year‐old female who presented to the general surgery department for complaints of abdominal pain for 1 h following blunt abdominal trauma. Pain was of mild to moderate intensity, located in the right lower region. Vitals were stable. Physical examinations revealed mild tenderness on deep palpation. Extended Focused Assessment with sonography in Trauma (EFAST) revealed minimal free fluid in the pelvis with cholelithiasis.

## 3 Methods

In view of continuous aching pain in the patient, a CT scan was ordered, which revealed a hypodense lesion in the pancreas and left Sartorius muscle. No solid organ or bowel injury was noted. The patient was kept under observation.

**FIGURE 1:**
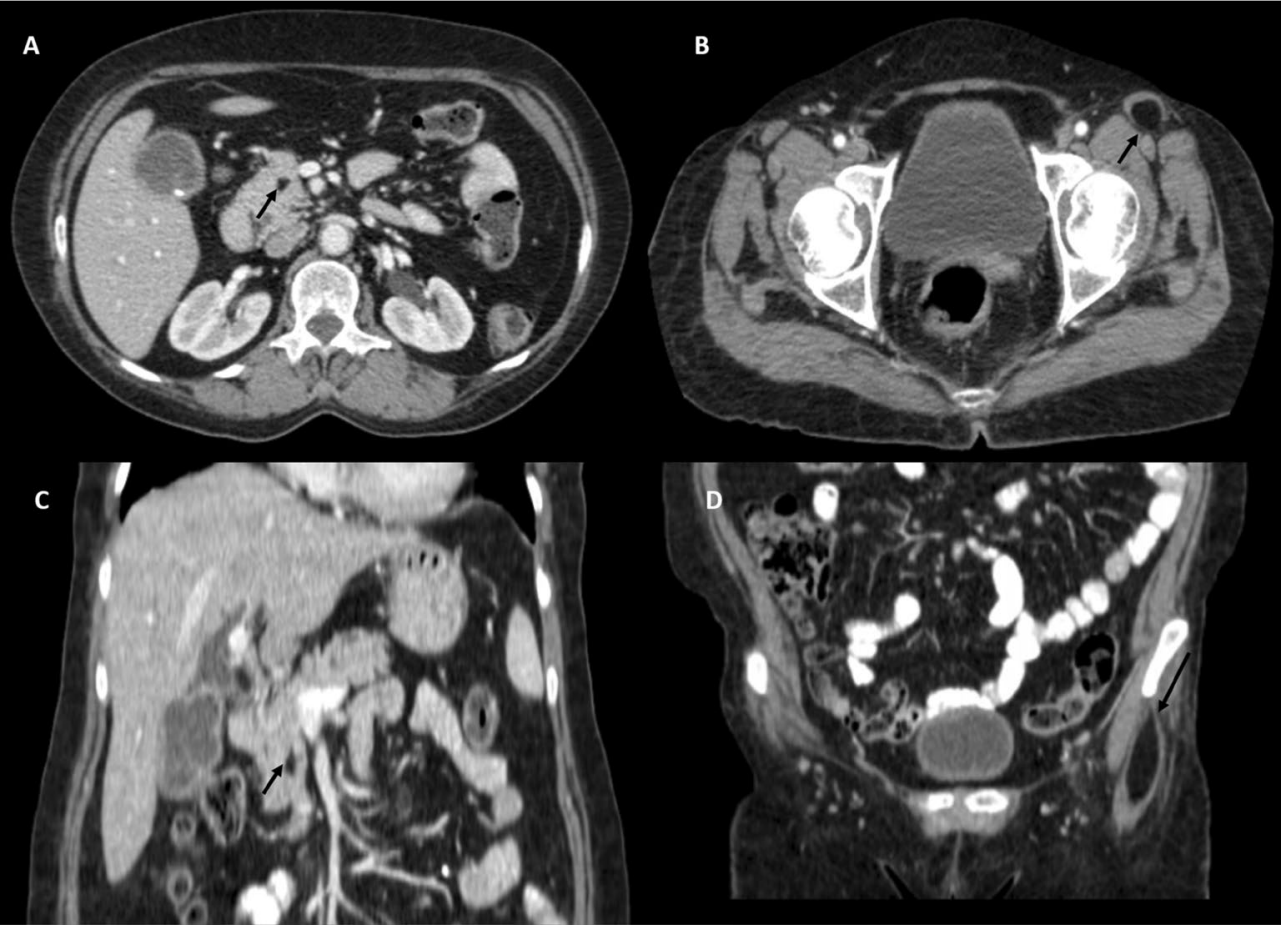
Axial (A, B) and Coronal (C, D) sequences of CECT abdomen showing a fat‐attenuating lesion in the proximal pancreatic head‐uncinate process junction (arrows) and left proximal sartorius (arrows) suggesting alipoma.

Contrast sections of the CT scan (Figure 1) of the abdomen showed a fat attenuating lesion (‐41HU) at the proximal head‐uncinate process junction of the pancreas measuring approximately 10 × 7 mm (craniocaudal × anteroposterior dimensions). Likewise, an incidental observation of an intramuscular fat attenuating lesion (‐44HU) was noted in the left proximal sartorius measuring approximately 5.4 × 1.8 cm (craniocaudal × transverse dimensions).

**FIGURE 2:**
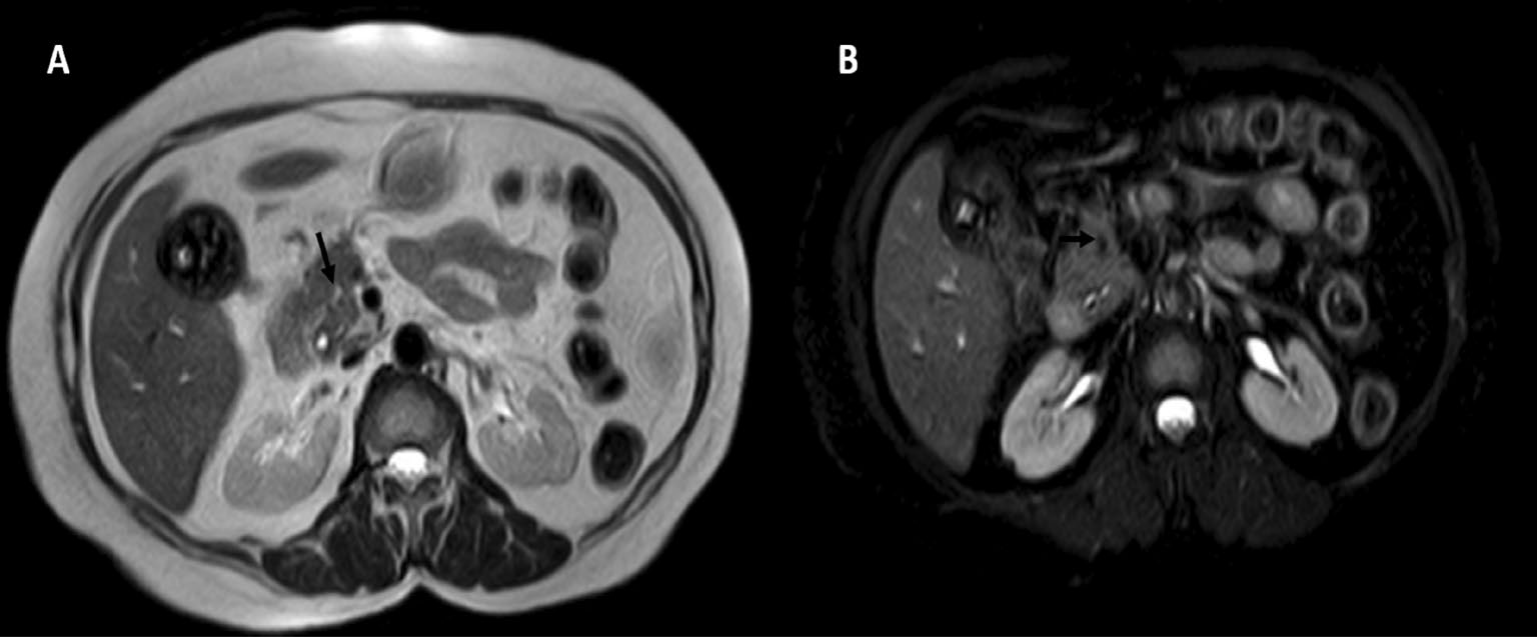
T2 non fat saturated axial (A) and T2 fat saturated axial (B) sequences showing a T2 hyperintense lesion in proximal pancreatic head showing suppression of signal on T2 fat saturated sequence suggesting lipoma (black arrow). Note is made of cholelithiasis.

MRI upper abdomen with Magnetic Resonance Cholangiopancreatography (MRCP) (Figure 2) was ordered in view of obscured CBD during USG evaluation, which revealed a T2 hyperintense focus showing loss of signal on fat‐saturated sequence suggesting lipoma. Common Bile Duct (CBD) was normal in caliber without any calculi.

## 4 Conclusion and Result(Outcome and Follow‐Up)

Patient was managed conservatively on Non‐steroidal anti‐inflammatory drugs (NSAID). Regarding lipoma, since it was asymptomatic, we kept our patient under observation, and our patient has not presented with any symptoms in the meantime.

In summary, this case report underscores the importance of considering lipomas in the differential diagnosis of well‐circumscribed, hypodense masses in various anatomical locations. The concurrent occurrence of pancreatic and sartorius muscle lipomas highlights the need for comprehensive evaluation and tailored management to ensure accurate diagnosis and effective treatment. Further studies may provide more insight into the etiological factors contributing to multiple lipoma formation and refine management strategies for such uncommon presentations.

## 5 Discussion

Pancreatic lipomas are exceedingly rare, with the first case reported by Bigard et al. in 1989 [2]. Mesenchymal tumors constitute 1%–2% of pancreatic tumors, and pancreatic lipomas account for less than 1% of all pancreatic tumors [1]. They are often discovered incidentally during imaging studies conducted for other conditions owing to their typically asymptomatic nature. In 2006, Hois et al. analyzed 6000 CT scans and identified pancreatic lipomas in 5 of them (0.083%) [3]. Pancreatic lipoma can range from 1 to 30 cm; in our case, it had a maximum dimension of 1 cm [4].

Sartorius muscle lipomas are also rare. Intramuscular lipomas may be challenging to diagnose because of their rarity, potential for an infiltrative growth pattern, and intermingling with muscle fibers. Intramuscular lipomas are relatively rare, comprising over 1.8% of all primary adipose tissue tumors and less than 1% of all lipomas. According to Fletcher et al. 83% of intramuscular lipomas are of the infiltrative type, while 17% are well defined. Although lipomas are multiple in 5%–15% of patients, most intramuscular lipomas are solitary and affect a single muscle [5, 6]. In most reported cases, lesions were found on the head of the pancreas, as in our case. Lesions in the tail and body of the pancreas have been reported in only a few instances.

The concurrent occurrence of lipomas in both the pancreas and the sartorius muscle in this patient is particularly noteworthy. The simultaneous appearance of lipomas in different body regions may suggest a systemic predisposition to lipoma formation or an incidental finding. No known genetic or environmental factors directly link these two conditions, but the possibility of a genetic syndrome or systemic condition influencing the development of multiple lipomas should be considered.

The clinical presentation depends on the site of origin, and patients typically present with a slow‐growing mass that is painless, non‐tender, and immobile. Although they may be asymptomatic, some lipomas may produce pancreatic or biliary obstruction or both. The clinical presentation of intramuscular lipomas is largely dependent on mass size. They typically manifest as slow‐growing asymptomatic swellings without a palpable mass. However, larger or deeper lipomas can compress or expand into the adjacent soft tissues or nerves, causing pain, which is a rare and late symptom [7, 8, 9, 10]. Nerve impingement can lead to paresthesia and neurological deficits in the area of nerve distribution. As the mass increases, patients may experience reduced range of motion or functional limitations due to mechanical restrictions. Symptoms can persist for months to years prior to diagnosis [11, 12]. In this case, the sartorius muscle lipoma was well‐circumscribed and homogeneous, consistent with typical lipoma characteristics, and did not display features suggestive of malignancy.

Although ultrasound is the initial imaging modality for most abdominal pathologies, its effectiveness in detecting pancreatic lipomas is limited, as they can appear hypo‐, iso‐, or hyperechoic. For sartorius muscle lipomas, ultrasound findings typically include a hyperechoic, well‐defined mass with fine internal echoes or a striated appearance due to interdigitation with muscle tissue [4, 13].

CT findings for pancreatic lipomas typically show hypodensity (−30 to −120 HU), homogeneity, and minimal contrast enhancement, with thin fibroreticular septa and no infiltration of surrounding tissues. For intramuscular lipomas, CT usually reveals a hypodense soft tissue mass with negative Hounsfield measurements, striated appearance, and interdigitations. The mass generally has an oval or fusiform shape. CT is a reliable method for distinguishing lipomas from liposarcomas. While liposarcomas in the abdominal cavity often present with large poorly defined areas of increased density, benign lipomas do not. However, well‐differentiated lipogenic liposarcomas may resemble benign lipomas on CT if they lack high‐density components. However, well‐differentiated liposarcomas tend to have atypical cells and thicker fibrous septa with some large and small blood vessels [14]. Endoscopic ultrasound‐guided fine‐needle aspiration (EUS‐FNA) can be useful for diagnosing unusual pancreatic masses and guiding treatment strategies, avoiding unnecessary surgery [4, 13].

MRI is considered superior to CT for preoperative planning. Lipomas are typically T1/T2 hyperintense and show suppression on fat suppressed sequences. For sartorius muscle lipomas, MRI typically reveals a fat‐containing mass within the muscle that is isointense to the subcutaneous fat across all sequences, sometimes with septa. These septa should not be significantly enhanced or show nodules. In infiltrative types, interlocked or intermingled muscular tissue and fibers may be observed. If a capsule is present outside the intramuscular lipoma, it should not contain muscular fibers within the main mass. For pancreatic lipomas, MRI shows hyperintensity on both T1‐ and T2‐weighted sequences, similar to intra‐abdominal and subcutaneous fats. Fat‐suppressed T1‐weighted images demonstrate homogeneous suppression of the signal intensity within the tumor.

Pathologically, lipomas are well‐circumscribed and nodular. The cut surface appears homogeneous with a fatty appearance. These tumors are usually small, measuring less than 5 cm. Histopathologically, a sartorius muscle lipoma is characterized by mature adipocytes arranged in a lobular pattern, typically enclosed by a thin fibrous capsule. The tumor exhibits uniform adipocytes with large lipid vacuoles, minimal inflammatory response, and absence of cellular atypia or increased mitotic activity. There are no documented cases of malignant transformation. Differentials for sartorius lipoma are liposarcoma, rhabdomyoma, rhabdomyosarcoma, fibromatosis, and neurofibroma. Likewise, pancreatic lipomas are also encapsulated in a thin collagen layer, which aids in surgical removal and differentiation from lipomatosis [15]. Fatty replacement, liposarcoma, and teratoma are other differentials. Liposarcoma is a notable differential as it requires surgery. Due to advancements and increased accuracy in imaging techniques, histopathological confirmation of pancreatic lipomas is seldom necessary. However, for larger tumors where distinguishing them from malignant lipid‐containing tumors can be challenging, histopathological evaluation may be required. In our case, no histopathological confirmation was obtained.

Simple observation is usually enough for asymptomatic cases, while surgical excision can be done for symptomatic cases and in cases of diagnostic uncertainty. While one study in particular has suggested aggressive follow‐up with USG in 6–12 months and CT scan in 2–5 years, other studies have not dictated the same. Short‐term interval observation has been advocated in some studies to prove stability to differentiate from liposarcoma. This variable practice of management is probably due to the absence of a written consensus regarding management [1, 16]. Regarding our case, we kept our patient on observation, and our patient has not presented with any symptoms in the meantime. Complications such as obstructive symptoms, including jaundice or pancreatitis, occur if they compress adjacent structures such as the bile or pancreatic ducts. Additionally, the radiographic appearance of pancreatic lipomas can mimic that of malignant tumors, potentially resulting in unnecessary invasive procedures or delays in appropriate management. Sartorius muscle lipomas can cause localized pain or discomfort due to pressure on surrounding muscles or nerves and may impair muscle function, affecting activities that involve hip flexion.

## Author Contributions

**Om Prakash Bhattaa:** data curation, formal analysis, investigation, software, supervision, writing – original draft, writing – review and editing. **Shritik Devkotab:** conceptualization, formal analysis, methodology, resources, writing – review and editing. **Arun Kalikotec:** data curation, formal analysis, investigation, software, writing – original draft, writing – review and editing. **Prakash Gyawalid:** conceptualization, formal analysis, investigation, supervision, validation. **Sachin Awasthie:** conceptualization, investigation, resources, validation.

## Ethics Statement

Patient anonymity is maintained and consent was obtained for publication from the patient.

## Consent

Written consent was taken from the patient for publication of this case and accompanying imaging findings.

## Conflicts of Interest

The authors declare no conflicts of interest.

## Data Availability

Anonymized data is accessible upon request from the corresponding authors.
